# Characteristics of pork belly consumption in South Korea and their health implication

**DOI:** 10.1186/s40781-015-0057-1

**Published:** 2015-06-09

**Authors:** Jee-Hwan Choe, Han-Sul Yang, Sang-Hoon Lee, Gwang-Woong Go

**Affiliations:** Department of Food Bioscience and Technology, Korea University, Seoul, 136-701 South Korea; Division of Applied Life Science Graduate School (BK21 plus), Gyoungsang National University, Jinju, 660-701 South Korea; Department of Nutritional Science and Food Management, Ewha Womans University, Seoul, 120-750 South Korea; Department of Food and Nutrition, Kookmin University, Seoul, 136-702 South Korea

**Keywords:** Pork belly, Pork preference, Pork belly production, Saturated fatty acids, Dyslipidemia, Cardiovascular disease

## Abstract

Fresh pork belly is a highly popular meat in South Korea, accounting for 59 % of the approximately 100 g of meat per capita that is consumed daily. Fresh pork belly offers not only high-quality protein from the lean cuts but also substantial micronutrients including fat-soluble vitamins and minerals. However, fresh pork belly generally consists of about 30 % fat, with saturated fatty acids representing half of this value. Excessive consumption of saturated fatty acids increases total cholesterol, low-density lipoprotein-cholesterol, and triglycerides while decreasing high-density lipoprotein-cholesterol, raising concerns about an increased risk of hyperlipidemia, followed by cardiovascular diseases. In this review, we discuss the consumption and production trends in South Korea, the general characteristics, and health issues related to fresh pork belly to delineate the features of pork production and consumer welfare.

## Introduction

**Fig. 1:**
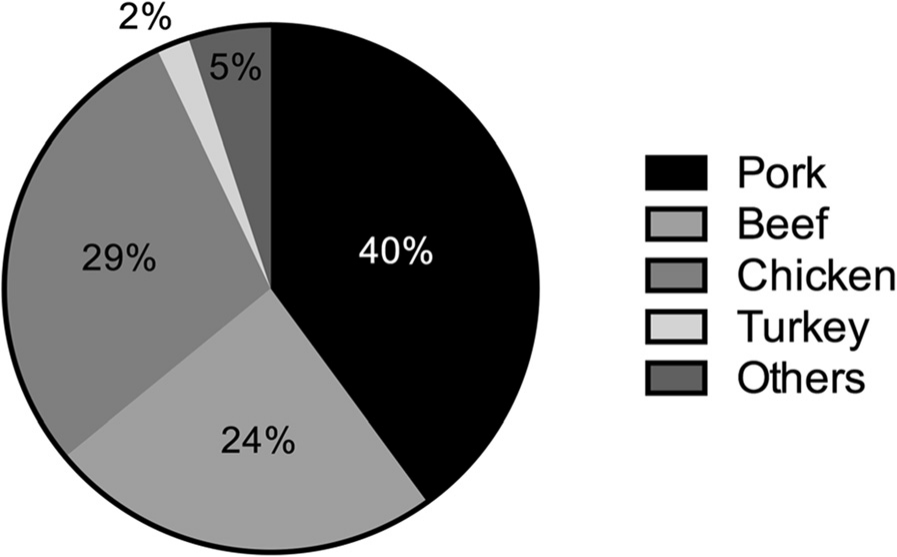
Meat consumption in the world. Data from report of Pork Checkoff (2012)

Pork is the most highly consumed meat in the world (Fig. 1) [1, 2], and global pork markets are expanding and becoming more competitive following recent bilateral free trade agreements [3]. South Korea is one of the highest pork-consuming countries in the world [2], and pork consumption in South Korea has steadily increased in recent years [4]. However, pork production in South Korea does not satisfy consumer demand [4, 5]. In addition, consumers in South Korea have a unique consumption pattern and a strong preference for high-fat cuts such as belly and Boston butt [5–7]. Pork belly (called Sam-gyeop-sal in South Korea) is the most favored primal cut among the various pork cuts. Therefore, the supply of pork belly depends on importation. In contrast, primal low-fat cuts such as loin, tenderloin, picnic shoulder, and ham (pork leg) face surplus inventory problems due to low consumer preference and exporting difficulties [5, 8].

Pork belly not only provides rich flavor and taste but is also a source of high-quality protein, vitamins, and minerals. However, it is also recognized that pork belly is the highest-fat cut among the various primal pork cuts, and therefore excessive consumption has potential adverse effects on humans, including increasing risk of cardiovascular disease and the metabolic syndrome [9–14]. Therefore, the goal of this article is to review the consumption and production of pork belly in South Korea and the general characteristics of pork belly and to discuss the potential harm to health of excessive consumption of the high fat in pork belly.

## Review

### Consumption and production of pork belly in South Korea

Pork consumption in South Korea is ranked seventh in the world and third in Asia (Table 1) [2]. In South Korea, pork carcasses are primarily cut into seven parts: Boston butt, picnic shoulder, loin, tenderloin, rib, belly, and ham. Among these primal cuts, belly is the most preferred part in South Korea, followed by Boston butt and rib [5, 8]. The common characteristic of these pork parts, especially pork belly, is a high fat content (Table 2). On the other hand, South Korean consumers have consumed less loin, tender loin, and ham cut, which are relatively low-fat containing parts. This preference is unique to consumers in South Korea [6, 7]. Indeed, United States consumers favor loin, Boston butt, and rib, and Japanese consumers prefer tenderloin, loin, Boston butt, and ham. Also, Chinese consumers favor pork ribs and loin [5].

**Table 1:** Top 10 countries in pork consumption per capita (kg)

Rank	Country	Year			
		2011	2012	2013	2014
1	Montenegro	45.1	46.7	38.6	44.6
2	China/Hong Kong/Macau	38.3	40.0	41.5	42.6
3	European Union	41.5	40.7	40.2	42.3
4	Serbia	38.0	38.6	39.5	40.0
5	Belarus	39.3	41.7	42.6	38.5
6	Taiwan	39.7	39.0	37.7	36.9
7	South Korea	29.9	30.9	32.4	32.9
8	Switzerland	32.5	31.6	32.6	32.8
9	United States	26.8	26.9	27.4	26.8
10	Norway	26.5	25.5	25.8	25.5

**Table 2:** Nutritional composition of different pork cuts (raw and cooked)^a^

	Energy (kcal/100 g)	Water (g/100 g)	Protein (g/100 g)	Fat (g/100 g)	Ash (g/100 g)	Carbohydrate (g/100 g)
Belly						
Raw	348	48.9	15.8	26.4	0.9	8.0
Roasted	493	32.7	21.9	41.1	1.0	3.3
Ham						
Raw	235	63.6	18.5	16.5	1.1	0.3
Roasted	299	45.7	38.2	14.1	0.8	1.2
Loin						
Raw	155	66.7	22.2	3.8	1.1	6.2
Roasted	242	57.9	27.3	13.9	1.4	0.0
Tender loin						
Raw	186	70.8	14.1	13.2	1.4	0.5
Roasted	220	53.4	40.3	5.3	1.0	0.0

^a^Data from food composition table of Rural Development Administration (2011)

**Fig. 2:**
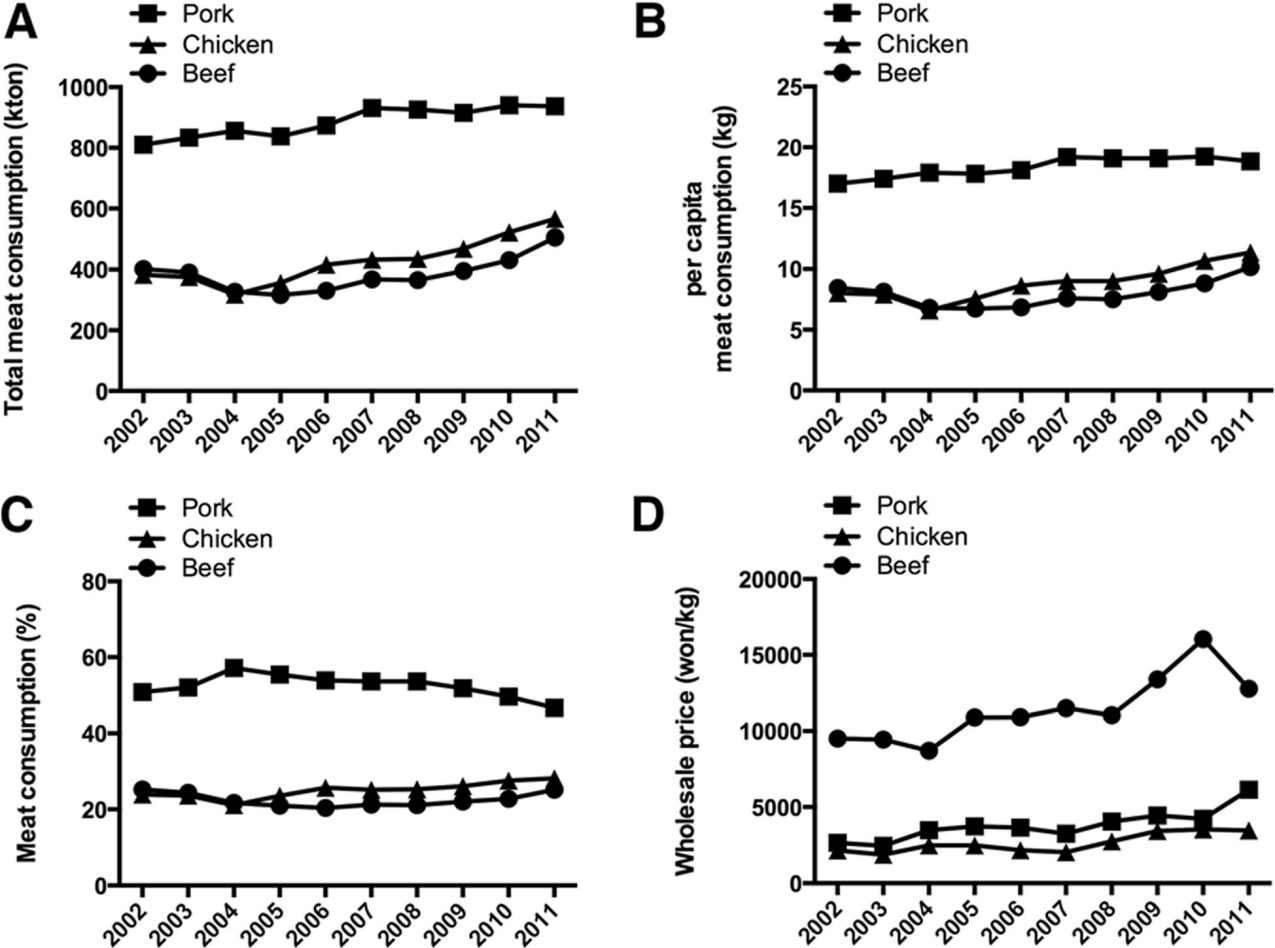
Meat consumption in South Korea from 2002 to 2012. **a** Total meat consumption; **b** Per capita meat consumption; **c** Meat consumption percentages; **d** Wholesale meat prices. Data from Korea Meat Trade Association

Meat consumption in South Korea has steadily increased, and pork has been the most highly consumed meat (Fig. 2a). Pork consumption, at 20.9 kg per capita (total 1049 kt), comprised almost half of the total meat consumption (42.7 kg per capita, total 2148 kt) in 2013, followed by chicken (11.6 kg per capita, total 580 kt) and beef (10.3 kg per capita, total 519 kt) (Fig. 2b and c) [4]. Interestingly, pork consumption in South Korea does not typically trend with pork price. Pork consumption (total pork consumption as well as per capita pork consumption) has slightly increased, although the wholesale pork price has also increased from 2002 to 2010 (Fig. 2d). Moreover, pork consumption remained relatively constant despite a sharp increase in the wholesale pork price from 2008 to 2010. From 2003 to 2008, the inventory rate of pork belly in South Korea was less than 30 % except 2005, but the inventory rates of other parts such as loin, picnic shoulder, and ham have always more than 30 %. A high inventory rate means there is more pork in inventory than is consumed by the customer. The lower inventory rate of pork belly indicates that customers in South Korea consumed more belly than other parts of the pig [5]. All of these facts imply that consumers in South Korea prefer pork belly far more than any other parts of the pig, resulting in considerable importation from other countries due to the severe imbalance in the supply and demand of pork belly.

**Fig. 3:**
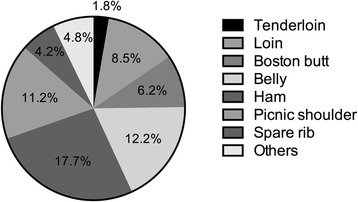
Average yield (%) of primal cuts from a pork carcass. Data from Korea Meat Trade Association

**Fig. 4:**
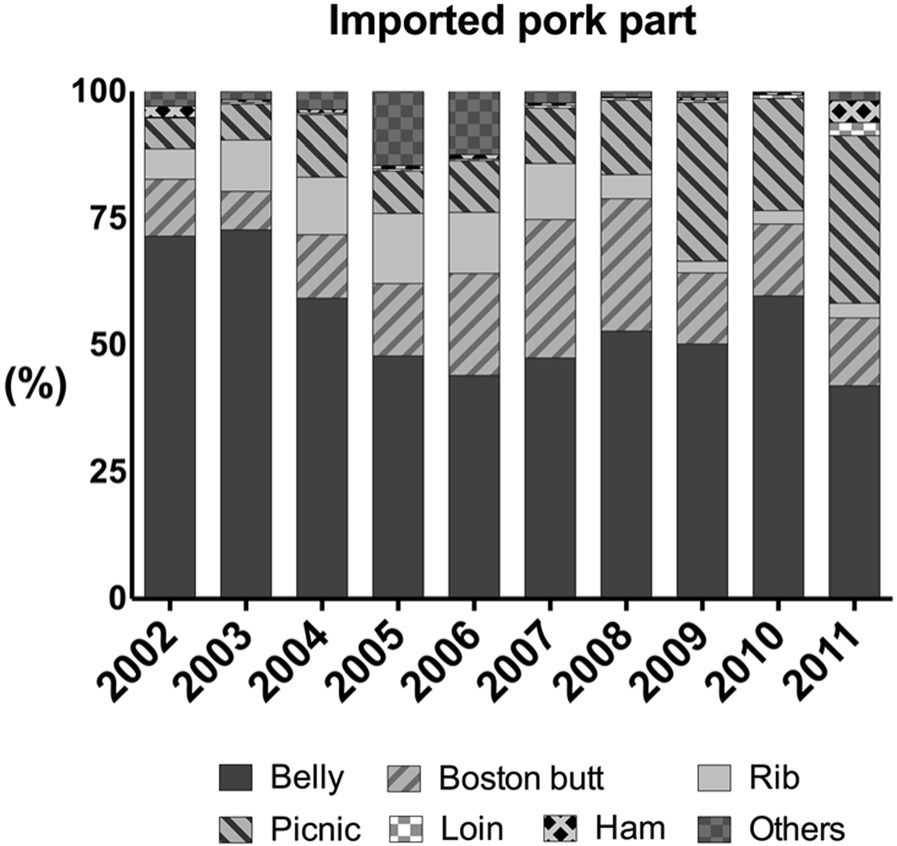
Changes in importation of pork in South Korea from 2002 to 2011. Data from Korea Meat Trade Association

Contrary to the strong preference and high demand for the high-fat cut in South Korea, pork belly (12.2 %) and Boston butt (6.2 %) are not a highest yield cut, comprising approximately 18.4 % of a pork carcass (Fig. 3). Low-fat pork cuts for which South Korean consumers have a low preference, comprise a higher proportion (about 37.4 %) of a pork carcass (e.g., ham 17.7 %, picnic shoulder 11.2 %, and loin 8.5 %) [4, 5, 15]. Consequently, though pork belly is not a low-yield cut, the immoderate preference for pork belly in South Korea has caused an imbalance in the supply and demand of pork belly. Moreover, outbreaks of foot-and-mouth disease (FMD) have a negative effect on South Korea’s pork industry since 2000. The small farming operations in South Korea have suffered from a reduced profit due to FMD and increased feed costs, resulting in a loss of small farming operations and an overall decrease in production efficiency [5]. Eventually, overall decrease in pork production efficiency have caused the price rise of pork belly. In addition, contrary to the South Korean market, the global supply of pork, especially the belly cut, is much higher than the global demand, so the South Korean pork market heavily depends on importation from other countries to meet the high domestic demand for pork belly (Fig. 4). The domestic supply rate of pork in South Korea have a tendency to decrease, from 92.8 % in 2003 to 72.8 % in 2013. Furthermore, pork belly comprise approximately 50 % of all imported pork [5]. Although the origin of pork is an important factor to consumers in South Korea, importation of pork belly is inevitable because of the severe imbalance in the pork market in South Korea [5].

### General features of pork belly fat for production in South Korea

Pork belly comprises approximately 12 % of chilled pig carcass, but represents approximately 15–17 % of the total carcass’s value (Fig. 3) [4, 15, 16], which means pork belly is an economically principal part of the pig. But, it is obvious that pork belly is an extremely fatty cut among the various primal cuts although an excessive fat content is not favorable to most of consumers and underlies the pathophysiology of many metabolic syndromes and chronic diseases. Previous study showed much higher fat content (approximately 40–50 %) of pork belly, which is regardless of factors such as genetic background, diet, sex, slaughter weight, and sampling location [17]. Similarly, all the pig breeds available in South Korea have over 30 % of fat in pork belly cut [18]. Consequently, consumer demand for leaner pork cuts has been increasing in many countries for years [19], resulting in a reduction in pork belly fat content of almost 29 % last 40 years [20, 21]. In sum, the most important characteristics of pork belly are associated with fat and include fat content and fatty acid composition.

Regarding the fat content of pork belly, large differences exist between breeds [22]. Two traditional pig breeds (Berkshire and Tamworth) have a higher fat content, thicker backfat, and less lean meat compared with two modern breeds (Duroc and Large White) [23]. Similarly, the belly cut from Berkshire pigs have a significantly higher fat content [18]. Furthermore, thinner backfat is generally correlated with lower fat content of the pork belly [24]. Genetic selection and/or cross-breeding also improved the growth rate and carcass composition with lower fat and higher lean content [25]. In addition, pig breeds (Landrace, Large White, Pietrain, and Hampshire) selected for leaner pork production have a superior carcass composition with lower fat and a higher lean content than traditional breeds (Tamworth, Saddleback, and Gloucester Old Spot) [22]. Besides, other factors such as sex, growth rate, and slaughter weight, influence the fat content of the pork belly. Barrows have a higher belly yield, thicker belly and backfat, and higher total fat content in their carcasses than gilts [26, 27]. Pigs with a fast growth rate and heavier slaughter weight also produce more belly portion and have higher fat content in the belly cuts compared with those with a slow growth rate and lighter slaughter weight [17, 23, 27–30].

Moreover, above factors influencing fat deposition exert modifications on fatty acid composition in pork belly [25]. Previous studies showed that higher fat deposition in the belly is observed in barrows compared to gilts and boars [24]. Moreover, higher fat deposition is generally associated with higher degree of fat saturation [31]. Thus, efforts for higher lean content in carcasses reduce fat deposition and increase fat unsaturation, resulting in a thinner and softer belly [21, 25]. The belly fat of gilts and slow-growing pigs contains a lower proportion of saturated fatty acids (SFAs) such as palmitic acid (C16:0) and stearic acid (C18:0), along with a higher proportion of linoleic acid (C18:2n-6), and of total polyunsaturated fatty acids (PUFAs). Consequently, the belly cuts from gilts and slow-growing pigs have higher PUFA/SFA ratio and n-6/n-3 ratios [21].

There is an interesting difference of perception about pork belly (or bacon) between Western countries and South Korea, because favored fatty acid profiles of pork belly are aiming different marketability. In Western countries, they primarily process pork belly as bacon, which is the cured and processed form of belly. On the contrary, consumers in South Korea favor grilled or roasted bellies rather than cured or processed bacon [32]. This difference led producers in each region to established distinct production strategy, i.e. Western consumer preferred higher saturation in pork belly. In fact, belly cut obtained from thick belly had the highest processing yields through the smoking and cooking processes. On the other hand, bacon from thin belly had the lowest slicing yields and a lack of crispiness [20]. During pork processing, a leaner belly with a lower degree of fat saturation is associated with problems related to increased handling, processing, and slicing difficulties; reduced bacon yield; and production of unattractive bacon [33, 34]. In particular, the firmness of the belly fat shows a strong positive correlation with the proportions of palmitic acid and stearic acid, whereas negative correlation with the proportions of linoleic acid, linolenic acid, and other PUFAs [21, 33, 35]. In addition, products with a high degree of unsaturated fats are more prone to rancidity during storage, resulting in reduced shelf-life [18, 21]. Based on these features, increasing unsaturation of pork belly is not desirable in Western countries. However, strategies for reducing fat content and increasing the degree of unsaturation in pork bellies does not affect marketability in South Korea.

### Benefits and risks of pork belly consumption

It is well known that pork meat provides not only high-quality protein from the lean cuts but also key micronutrients including fat-soluble vitamins and minerals. Thus, it is commonly accepted that meat provides a means for reducing malnutrition and increasing food security in developing countries [9, 36]. However, in developed countries where high fat and excessive calories are regularly consumed, meat consumption may underlie the pathophysiology of non-communicable diseases including cardiovascular disease, obesity, dyslipidemia, and cancer [9–14]. Indeed, high fat intake through consuming red meat such as fresh pork belly likely accelerates these adverse health conditions. Controversy exists as to the role of red meat consumption in the increased risk of developing public health-related diseases. Interpretation of results from prospective cohort studies has created uncertainty about the role of animal fat in the development of atherosclerosis and cardiovascular disease [37]. Nonetheless, numerous researchers have reported that colon cancer and cardiovascular disease are highly associated with excessive red meat consumption [10–12, 38]. The relationship between a high consumption of fat, a significant feature of pork belly, and dyslipidemia and cardiovascular disease is largely undisputed.

Unprocessed pork belly contains approximately 48 % fat and 39 % lean content [32]. Fat content is, in general, greatest in the dorsal portion of the belly and lowest in the ventral portion. The most abundant fatty acids in pork belly are monounsaturated fatty acids (MUFAs) followed by SFAs and PUFAs (47 %, 36 %, and 16 %, respectively) [21]. It is the proportions of specific fatty acids in the diet that are associated with the causes and prevention of coronary heart disease (CHD) rather than the total amount of fat [39–44]. It is particularly evident that there is a strong association between the incidence of CHD and SFAs or foods containing SFAs such as red meat. When SFAs in the diet are replaced by MUFAs or PUFAs, the risk of CHD is significantly reduced [45, 46]. SFAs are known to elevate the low-density lipoprotein (LDL)/high-density lipoprotein (HDL) ratio, potentiating foam cell formation and atherosclerosis [42, 47, 48]. In addition, after the American Heart Association (AHA) recommended decreasing SFA intake in 1961, there was a dramatic decline in CHD in Western countries [49]. It is noteworthy that replacing SFAs with MUFAs or PUFAs more successfully reduces the incidence of CHD than simply reducing total fat consumption [47]. Likewise, the LDL-cholesterol level and the total cholesterol/HDL-cholesterol ratio were reduced when SFAs were substituted with MUFAs. The PUFA/SFA ratio is an important indicator of CHD with a lower PUFA/SFA ratio correlating with a greater risk of CHD [45]. Fresh pork belly has a PUFA/SFA ratio of 0.48 and an n-6/n-3 ratio of 17.98. Values of 0.45 or above for the PUFA/SFA ratio and 4.0 or below for the n-6/n-3 ratio have been recommended in the United Kingdom.

The Korean Dietary Reference Intakes recommend that energy from fat should not exceed 25 % of total daily intake. These guidelines also suggest that saturated fat intake should not exceed 4.5–7.0 % and total cholesterol intake should be less than 300 mg/day for adults [50]. Additionally, according to AHA guidelines, the SFA intake should be limited to 7–10 % of daily calories [51]. However, approximately 48 % fat in 100 g of fresh pork belly contains 441 calories, which certainly exceeds the guidelines in general. Koreans consumed 24 g of pork belly per day during 2011, resulting in the intake of 11.5 g (104 calories) of fat, 4.1 g (37 calories) of SFAs, and 17.3 mg of cholesterol from this meat alone [4]. Furthermore, if these calculations are adjusted for age, there is little doubt that Korean adults consume a significant amount of fresh pork belly, which will increase the risk of non-communicable diseases. Approximately 100–200 g of fresh belly meat alone will exceed the guideline limits of SFA, cholesterol, and total fat intake. Therefore, most health organization guidelines limit red meat consumption chiefly to aid in reducing SFA and cholesterol consumption. In conclusion, excessive consumption of pork bellies as part of an unbalanced diet is highly likely to lead to impaired nutrient intake and abnormal fatty acid profiles, thereby negatively affecting long-term health.

### Changes in pork consumption patterns in South Korean households

As mentioned previously, the strong preference and high demand for pork belly led to an imbalance in the supply and demand of pork production in South Korea. These imbalances increased the price of pork belly and depreciated the relative economic value of other parts of the pig. These trends are clearly not viewed as progress in the pork industry. The data from Statistics Korea in Table 3 clearly show that, although pig productivity has been slightly improved due to increase in the number of post-weaned piglets per sow per year (PSY) and the number of marketed pigs per sow per year (MSY), pig production cost per 100 kg live weight have been greatly increased from 2008 to 2012 (exception for 2011 due to FMD outbreaks), leading to decrease in net income. Because pork belly is the most consumed pork part, pork producers shift the loss in net income into pork belly price, resulting in pork price have been increased continuously. It has become more difficult that consumers in South Korea consume the pork belly due to too much expensive. Furthermore, research has indicated that excessive consumption of fat, including consumption of pork belly, threatens public health in South Korea. Taken together, the most desirable shifts in pork consumption would be to decrease that of pork belly and to increase that of other leaner cuts.

**Table 3:** Pig production cost per 100 kg live weight and pig productivity

	Year					
	2008	2009	2010	2011	2012	2013
Production cost^a^	221,893	238,748	247,783	302,231	293,577	290,094
Net income^a^	59,542	88,281	40,389	143,455	9,139	−27,950
PSY^b^	19.8	20.5	20.1	19.4	20.1	20.8
MSY^c^	17.3	18.1	17.8	17.6	18.1	19.3

^a^Korean won

^b^Number of post weaned piglet per sow per year

^c^Number of marketed pigs per sow per year

As seen in Table 4, a recent survey showed a meaningful change in the purchase patterns of pork cuts in South Korean households (2015 agricultural forecast from the Korean Rural Economic Institute). From 2011 to 2014, the proportion of pork belly purchased among the pork cuts decreased from 36.5 % to 32.2 %, whereas that of picnic shoulder and ham increased by 2.5 % and that of loin and tenderloin increased by 1.1 %. In addition, according to this report, these tendencies are not temporary and consuming low-fat primal cuts should continue to be promoted not only for consumer health reasons but also for stabilization of the pork industry.

**Table 4:** Changes in purchase patterns of primal pork cuts in South Korean households

Cut of meat	Year			
	2011	2012	2013	2014^a^
Belly	36.5 %	33.9 %	31.8 %	32.2 %
Boston butt	12.5 %	12.6 %	13.0 %	12.2 %
Rib	6.0 %	6.6 %	7.3 %	6.5 %
Picnic shoulder and ham	21.0 %	23.4 %	23.8 %	23.5 %
Loin and tenderloin	4.7 %	4.5 %	5.2 %	5.8 %
Other	19.4 %	19.0 %	19.0 %	19.8 %

^a^Data were collected from January to November 2014

## Conclusions

Pork is the source of high-quality protein, vitamins, and minerals. Consumers in South Korea also favor pork rather than chicken and beef. Especially, they strongly prefer pork belly, the highest fat and the lower yield cut. The unique and strong consumption pattern in South Korea caused severe imbalance between demand and supply of pork belly, resulting in heavily depend on import from foreign countries. In addition, excessive consumption of pork bellies as part of an unbalanced diet is highly likely to lead to impaired nutrient intake and abnormal fatty acid profiles, thereby negatively affecting long-term health. These implies that preference for pork belly in South Korea have potential risk to domestic pork industry development and consumers health. However, the meaningful change is recently observed that purchase of belly decreased and consuming low-fat primal cuts increased. These shifts in pork consumption can help not only stabilization of the pork industry but also consumer welfare in South Korea.
